# Short inter-pregnancy interval: why is it still high among women in Dar es Salaam?

**DOI:** 10.11604/pamj.2021.40.17.29770

**Published:** 2021-09-06

**Authors:** Amani Idris Kikula, Andrea Barnabas Pembe, Bruno Sunguya

**Affiliations:** 1 Department of Obstetrics and Gynaecology, Muhimbili University of Health and Allied Sciences, Dar es Salaam, Tanzania,; 2 School of Public Health and Social Sciences, Muhimbili University of Health and Allied Sciences, Dar es Salaam, Tanzania

**Keywords:** Pregnancy, contraception, maternal health, pregnancy outcome, maternal mortality

## Abstract

**Introduction:**

in Tanzania, for the past decade, there has been a rising trend of women with short inter-pregnancy interval (IPI) (16% to 19%). Short IPI is associated with poor maternal and neonatal outcomes. We aimed to determine the factors associated with short IPI among women attending antenatal clinic (ANC) at Mnazi Mmoja Hospital, Dar es Salaam, Tanzania.

**Methods:**

a cross-sectional study was conducted in September 2018 at Mnazi Mmoja hospital among women receiving ante-natal care. A total of 530 women were included in the analysis. Analysis was conducted through SPSS version 24 computer program using descriptive analyses to determine the IPI and characteristics thereof, and logistic regression analysis to examine factors associated with IPI among pregnant women. Associations with a p-value < 0.05 were considered statistically significant.

**Results:**

twenty-two percent of the women attending ANC in Mnazi Mmoja hospital had short IPI. Short IPI was associated with young (<25years) age (AOR=2.67, 95% CI=1.23-5.79); non-use of a contraceptive method (AOR=2.05, 95%CI=1.22-3.45); breastfeeding for less than 6 months (AOR=3.45, 95% CI=1.17-10.13) and having an antecedent dead child at the time of index conception (AOR=3.38, 95% CI=1.15-9.93).

**Conclusion:**

about 1 in every 5 women attending ANC in Dar es Salaam had a short IPI. Addressing short IPI will complement the government´s efforts to improve maternal indicators in Tanzania and areas with similar contexts. Such efforts should emphasize in adherence to recommended infant feeding practices, women at a younger reproductive age group, those with a history of pregnancy loss, and strengthening contraception use among women of reproductive age.

## Introduction

Inter-pregnancy Interval (IPI) is the duration a woman spends not being pregnant before the index pregnancy [1]. The World Health Organization (WHO) recommends at least 24 months and not more than 59 months following a live birth, to avoid adverse pregnancy outcomes [1]. Short IPI has been associated with several adverse maternal outcomes including post-partum hemorrhage, premature rupture of membrane, hypertensive disorders of pregnancy, preterm labor and maternal mortality [2-5]. While in newborns, it is associated with low birth weight and perinatal death, among others [6-8]. Two hypotheses have explained the reasons for the outcomes of short IPI. First, the continued nutritional drainage with short IPI diminishes maternal nutrients ending up in starting to use maternal nutritional reserves at the beginning of pregnancy. This is termed as the “maternal nutritional depletion hypothesis [9]. Parallel to this, with the shortened recovery time to the mother through pregnancy and lactation episodes which worsens the status (body nutrient reserve) even more, mostly recovery of folate reserves folate depletion hypothesis [10]. The two states act as a continuous vicious cycle of maternal ill-health among those with short IPI. Thus, both of the above situations subject a woman to a compromised pre-conception nutritional state to support the index pregnancy to term without complications [11].

The Government of Tanzania has improved the availability and accessibility of family planning services over the years. This is by improving the ministry of health's annual budget allocation [12]. This has resulted in an increase in the use of modern contraceptives, from 27% to 32% over the past decade. However, with a slow decline in total fertility rate, from 5.7 to 5.2. Despite these efforts and achievements, the proportion of women with short IPI has increased from 16% to 19% over the same period [13]. Local studies have shown an even higher magnitude, ranging from 19% to 48% [2, 6, 7]. The proportion of women in Tanzania with short IPI is still high despite the interventions in existence such as improving access and availability of family planning methods for child spacing. Various factors may influence it including socio-demographic characteristics, psychological, and obstetric factors. Adherence and correct use of available family planning methods may also be associated with IPI in a context like Tanzania, though the evidence is not available. This study, therefore, aimed to determine the magnitude of IPI and factors associated thereof among pregnant women attending antenatal clinic in Dar es Salaam, Tanzania. Understanding the interaction of these factors with the IPI will help towards improving maternal and perinatal health using the minimal available resources.

## Methods

**Study design and area:** this cross-sectional study was conducted in September 2018 among pregnant women attending the antenatal clinic (ANC) at Mnazi Mmoja Hospital in Dar es Salaam, Tanzania. The hospital is at the city center, with easy accessibility through public transportation from all areas of Dar es Salaam. It is also one of the oldest centers providing antenatal clinic care in the city. Women attending this clinic come from all areas of the city making the facility ideal for a representative population.

**Study sample:** all women in their ANC booking and follow-up visits with a previous live birth that lasted at least 28 weeks of gestation age were included for data collection. Those with communication impairment and severe systemic illness were excluded. The sample size of 524 was calculated from Open Epi program for descriptive study; where, n = the minimum sample size, z= level of confidence (95%), z = 1.96, ε = maximum likely error = 5%, D = design effect, f= non response rate adjustment and P = prevalence of short IPI in Tanzania = 19% [13]. With an assumption that the population attending Mnazi mmoja antenatal clinic is from 5 clusters of Dar es Salaam administrative districts, a design effect of 2 was considered.

**Variables and tools:** the outcome variable was IPI that was calculated using the last normal menstrual period or date of an early ultrasound to extrapolate the LNMP and birth date of the antecedent pregnancy [14]. For this study, it (short IPI) was operationalized to mean the duration in months after a previous live birth to the last normal menstrual period prior to the index pregnancy which is less than 24 months. Independent variables included marital status categorized as married or living with someone and not married or not living with someone; maternal age collected in years then further categorized into < 25, 25 - 29, 30 - 34 and ≥ 35; religion status categorized as Christian or Muslim, which are the major religions in the country; parity status categorized as para 1 or multipara; child sex preference categorized as a male or female child; education level categorized as no formal education, at least primary school level and at least secondary school level; mode of previous delivery to vaginal delivery and operative delivery. The tool for the independent variables was adapted from the TDHS 2015-2016 questionnaire to meet the objectives of this study [13].

Knowledge on contraception was dichotomized into adequate knowledge and inadequate knowledge. A composite score was used to group the knowledge levels. One with at least three questions correct defined adequate knowledge [15]. Intention to get pregnant had two groups; 1) intended scored as; if the woman stopped using contraception or had not used contraception because she wanted to become pregnant and the pregnancy occurred at about the right time in her life, then the pregnancy was intended (with a score of 3), and 2) unintended: this model was adopted from the national health of statistics report and modified to suit this study [16]. The questionnaire was initially translated to Swahili (following compilation of the questions) then back-translated to English to ensure retention of the meaning of all the questions. Then it was pretested at the same facility before the initiation of data collection from which no changes to the questionnaire were deemed necessary.

**Data collection:** upon arrival at the hospital, women submitted their cards to the nursing station. The submitted antenatal cards were sorted by the principal investigator as per inclusion criteria and labeled with a blue marker dot (to avoid re-recruitment on subsequent visits). Attending nurses were instructed on the labeling on the cards, to direct women with the marked cards to the research team after they have been attended to. The first eligible client was randomly selected from the labeled cards (follow-up clients) and for booking clients, the first client was randomly selected from the registry. The subsequent clients were consecutively recruited until the sample size was achieved. All women received antenatal care as per institution protocol. The principal investigator randomly asked women after they were seen by the attending health care worker to ensure a comprehensive daily inclusion. For those who consented, data was collected via an interview using a structured Swahili questionnaire by the principal investigator and pre-trained research assistants (recently graduated medical doctors). This was done from Monday to Friday, morning until all the clients were attended to avoid selection bias.

**Data analysis**: using SPSS version 24, coded data were entered daily. Data cleaning was done sequentially via manual checking entered data and cross tabulating the entered variables before analysis until the completion of data entry. The dependent variable was recoded to a binary variable of short and normal IPI coded as 1 and 0 respectively. Analyses were conducted using descriptive and regression methods. The description of the participant's characteristics was done through frequency distribution tables then the difference between categorical variables was determined using a chi-square test. The variables were put for logistic regression analysis, first with univariate logistic regression, from which those with p-value < 0.2 were subjected to a multivariable logistic regression model to determine the level of effect of the independent variables to the dependent variable with a P-value less than 0.05 considered statistically significant. The magnitude of effect of the independent variable to the dependent variable was estimated using an odds ratio at a 95% level of confidence.

**Ethical considerations:** the Senate Research and Publication Committee of Muhimbili University of Health and Allied Sciences (MUHAS) gave ethical clearance for the performance of the study (reference number DA.287/298/01A). Permission to conduct the study was also obtained from the offices of Ilala district medical officer, director of hospital services and the nurse in charge of the reproductive and child health unit of Mnazi Mmoja Hospital. Written informed consent was obtained from all the study participants. The right to withdraw from the study any time they wished was granted and withdrawal from the study did not affect their subsequent antenatal and postnatal care or other services. Study participants´ names were not used in the study. Other antenatal care services were performed as per institution protocol.

## Results

There were 1,397 women attending antenatal care during the study period of whom 873 were not included in the analysis 103 (refused participation), 512 (primigravida), 101 (had second or more visits) and 151 (had an abortion in a previous pregnancy). A total of 530 women were included in the study. Among 530, 6 were excluded in the analysis due to incorrectly filled questionnaires (inclusion of women with abortion and with no date of birth of the antecedent child), thus remaining with 524 in the study for analysis. The proportion of women with short IPI was 22.14%. The mean age of women studied was 29.80(±4.94). Over 9 in 10 women were married or living together with a partner and had at least a primary level education. Only a third of the women had adequate knowledge of contraceptives and 75% did not use a method of contraception prior to index conception. However, only 1 in 20 women intended to get the index pregnancy. While over 40% of the women had no preference for the sex of the child. The majority had a live antecedent child at the time of index conception and breastfed for at least 6 months in Table 1. Women aged less than 25 years had more than twice the odds for having short IPI than those with 30-34 years. While those who did not use contraceptives had twice the odds for short IPI than those who used. Breastfeeding for less than 6 months tripled the odds of having short IPI and those who had a dead antecedent child were more than three likely to have short IPI than those with a live antecedent child in Table 2.

**Table 1:** distribution of women per their socio-demographic characteristics (N = 524)

Characteristics	Inter-pregnancy interval			
		Short IPI n=116	Normal IPI n=408	P-value
	Total	n (%)	n (%)	
**Maternal Age**				
<25	86	32(37.21)	54(62.79)	**0.000**
25-29	167	40(23.95)	127(76.05)	
30 - 34	177	28(15.82)	149(84.18)	
≥35	94	16(17.02)	78(82.98)	
**Marital Status**				
Married/Living together	483	106(21.95)	377(78.05)	0.717
Not Married/Not living together	41	10(24.39)	31(75.61)	
**Education Level**				
No formal education	8	2(25.00)	6(75.00)	0.642
At least primary education	283	65(22.97)	218(77.03)	
At least secondary education	233	49(48.27)	184(51.73)	
**Occupation**				
No employment	141	30(21.28)	111(78.72)	0.723
Self-employment	329	76(23.10)	253(76.90)	
Formal employment (gov/private)	54	10(18.52)	44(81.48)	
**Child sex preference**				
Yes	219	43(19.63)	176(80.37)	0.242
No	305	73(23.93)	232(76.07)	
**Intention for pregnancy**				
Yes	30	4(13.33)	26(86.67)	0.232
No	494	112(22.67)	382(77.33)	
**Knowledge on contraceptive methods**				
Knowledgeable	166	38(22.89)	128(77.11)	0.777
Not Knowledgeable	358	78(21.79)	280(78.21)	
**Contraceptive use prior to index pregnancy**				
Used at-least one contraceptive method	388	61(15.72)	327(84.28)	**0.000**
Did not use any contraceptive method	136	55(40.44)	81(59.56)	
Mode of delivery				
**Vaginal delivery**	401	91(22.69)	310(77.31)	0.580
**Operative delivery**	123	25(20.33)	98(79.67)	
**Parity status**				
Para 1	274	69(25.18)	205(74.82)	**0.079**
Multipara	250	47(18.80)	203(81.20)	
**Duration of breastfeeding**				
<6 months	78	53(67.95)	25(32.05)	**0.000**
≥6 months	446	63(14.13)	383(85.87)	
**Outcome of previous pregnancy**				
Alive and well	444	62(13.96)	382(86.04)	**0.000**
Died	80	54(67.50)	26(32.50)	

**Table 2:** multivariable logistic regression analysis for the odds of short inter-pregnancy interval (N = 524)

	Short IPI	Bivariate analysis		Multivariable analysis	
Characteristic	n(%)	COR	95% CI	AOR	95% CI
**Maternal age**					
<25	86(16.41)	3.15	1.74 - 5.72	2.67^*^	1.23 - 5.79
25 - 29	167(31.87)	1.68	0.98 - 2.87	1.63	0.86 - 3.09
30 - 34	177(33.78)	1			
≥35	94(17.94)	1.09	0.56 - 2.13	1.02	0.49 - 2.14
**Use of the contraceptive method**					
Used at-least one method	388(74.05)	1			
Did not used any method	136(25.95)	3.64	2.35 - 5.64	2.05^*^	1.22 - 3.45
**Parity status**					
Para 1	274(52.29)	1.45	0.96-2.21	0.77	0.44-1.37
Multipara	250(47.71)	1			
**Duration of breastfeeding**					
<6 months	78(14.89)	12.89	7.47-22.23	3.45^*^	1.17 - 10.13
≥6 months	446(85.11)	1			
**Status of the antecedent child at a period of index conception**					
Alive and well	444(84.73)	1			
Died	80(15.27)	12.80	7.46-21.94	3.38^*^	1.15 - 9.93

* - statistically significant at p<0.05

## Discussion

Among the women included in the study, about one-fifth of them had a short IPI. The occurrence of short IPI was independently associated with young maternal age (age less than 25 years), non-use of a contraceptive method prior to index pregnancy, short duration of breastfeeding and having a dead preceding child (of antecedent pregnancy) at a period of index conception. This study showed almost one in five participants had short IPI which does not differ from the national figure (19%) [13]. This could be justified by the existing tradition of considering every child comes with its blessing from God necessitating having many children during the reproductive period, hence short IPI [17]. In Nigeria, the magnitude was three times these findings [18]. The difference observed could be due to the study being done at a referral facility accommodating mostly high-risk women of Nigeria. Meanwhile, for the community-based methodological approach, the magnitude was also still high [7, 19]. Despite the variations, the magnitude of short IPI is still high in the study population, which warrants intervention.

Women who were aged less than 25 years had almost 3 times more odds than those who aged 30 to 34 years, to have short IPI. This could be explained by the practice of early marriage in Tanzania allowing them to achieve an adequate family size earlier. Eventually making them to have an adequate or longer spacing interval with advanced age. This stresses the need to intervene in this vulnerable group to improve their overall reproductive health. This is similar to studies done elsewhere in Africa [7, 20, 21]. However, this is contrary to the situation in developed countries [22, 23], where probably the culture of women attaining their career goals before starting a family is in place to eventually give older women a shorter IPI. Non-use of contraceptives was twice as much more associated with short IPI than its usage. This is magnified by having over two-thirds of the study sample having inadequate knowledge of contraceptive methods. The contraceptive use protective effect towards short IPI is similarly demonstrated in other studies [19, 24-26]. Furthermore, being compounded by the male-centered practice which requires male partner approval for use of a contraceptive method [27]. This illustrates the need to address not only the provision of contraceptives but educational and cultural circumstances surrounding contraceptive use.

Women who breastfed their children for less than 6 months were three times more likely to have short IPI compared to their counterparts. This could be due to the early return of fertility from loss of the physiological inhibitory influence of lactation towards the hypothalamus [28]. But also, possibly due to, over three-quarters of women studied did not use a contraceptive method prior to conception leading to a short IPI, necessitating them to possibly stop breastfeeding after knowing they are pregnant. Several other works of literature depict a similar picture [29, 30]. The breastfeeding contraceptive effect is not as efficient beyond 6 months post-partum [19, 20, 31], though it still proves a beneficial tool for having adequate spacing, among other advantages. Encouraging delivering women to exclusively breastfeed for at least 6 months could add an adequate spacing interval to the already nutritional benefit of the neonate. Those having a dead child at the time of conception had triple the odds of having a short IPI than those who had a live one. This could be due to the desire by the couple to replace the lost child after the mortality event, termed as the replacement theory [32]. This is further multiplied by the absence of the lactation contraceptive effect. This is similar to other studies done in developed and developing countries [21, 31, 33-35]. This qualifies the high-risk status for those with a dead antecedent child to warrant adequate healthy-spacing-counseling.

Despite the reported results, this study had some limitations. There was a possibility of social desirability bias while interviewing the study participants; this was mitigated partly by using validated tools especially on the knowledge of contraceptive methods and intention for pregnancy variables and assuring anonymity of the information provided. And it being a hospital-based study, it possibly took fairly more at-risk representative population, a community based, qualitative study design to explore the factors observed will bring more light into this. However, another strength of this study includes a focus on women with an antecedent livebirth excluding those with adverse birth events (abortion and stillbirths) which have different needs and expectations for future conception, eventually affecting IPI. Thus, giving a direction for this particular group of women and forming a stepping stone towards further understanding of how to overcome the high magnitude of short IPI in our community. Despite the stated limitations, the results are valid and can be a reflection of other similar urban hospital setup populations. Resources aimed at improving IPI in Tanzanian women should be focused onthe identified factors.

## Conclusion

One in every five women attending Mnazi Mmoja hospital had a short IPI. Addressing short IPI will complement the government´s efforts to improve maternal indicators in Tanzania and areas with similar contexts. Such efforts should be put to emphasize adherence to recommended infant and young child feeding practices, women at a younger reproductive age group, those with a history of pregnancy loss, and strengthening contraception use among women of reproductive age.

### What is known about this topic

- The magnitude of women with short IPI in Tanzania increasing over time;
- The proven adverse maternal and neonatal outcomes for women with short IPI.

### What this study adds

- The high magnitude of women with short IPI requires interventions to improve the overall maternal wellbeing in Tanzania;
- The socio-demographic risk factor associated with short IPI is- Younger age group of women and Obstetric risk factors associated with short IPI include - Women with a history of pregnancy loss prior to conception, women not using contraception prior to conception and those breastfeeding less than 6 months.
